# Persistent Decreases in Adult Subventricular and Hippocampal Neurogenesis Following Adolescent Intermittent Ethanol Exposure

**DOI:** 10.3389/fnbeh.2017.00151

**Published:** 2017-08-14

**Authors:** Wen Liu, Fulton T. Crews

**Affiliations:** Bowles Center for Alcohol Studies, University of North Carolina at Chapel Hill Chapel Hill, NC, United States

**Keywords:** adolescent intermittent ethanol, neurogenesis, hippocampal dentate gyrus, subventricular zone, rat

## Abstract

Neurogenesis in hippocampal dentate gyrus (DG) and subventricular zone (SVZ) matures during adolescence to adult levels. Binge drinking is prevalent in adolescent humans, and could alter brain neurogenesis and maturation in a manner that persists into adulthood. To determine the impact of adolescent binge drinking on adult neurogenesis, Wistar rats received adolescent intermittent ethanol (AIE) exposure (5.0 g/kg/day, i.g., 2 days on/2 days off from postnatal day, P25–P54) and sacrificed on P57 or P95. Neural progenitor cell proliferation, differentiation, survival and maturation using immunohistochemistry was determined in the DG and SVZ. We found that AIE exposure decreased neurogenesis in both brain regions in adulthood (P95). In the DG at P57, AIE exposure resulted in a significant reduction of SOX2+, Tbr2+, Prox1+ and parvalbumin (PV)+IR expression, and at P95 decreased DCX+ and PV+IR expression. AIE exposure also reduced the expression of two cell proliferation markers (Ki67+ and BrdU+IR with 300 mg/kg, 2 h) at P95. The immune signaling molecule β-2 microglobulin+ and the cell death marker activated caspase-3+IR were significantly increased in the DG by AIE exposure. In the SVZ, AIE exposure decreased SOX2+, Mash1+, DCX+ and Dlx2+IR expression at P95, but not at P57. Thus, in adulthood both brain regions have reduced neurogenesis following AIE exposure. To assess progenitor cell survival and maturation, rats were treated with BrdU (150 mg/kg/day, 14 days) to label proliferating cells and were sacrificed weeks later on P95. In the hippocampus DG, AIE exposure increased survival BrdU+ cells which differentiated into Iba1+ microglia. In contrast, SVZ had decreased BrdU+ cells similar to decreased DCX+ neurogenesis. These data indicate that AIE exposure causes a lasting decrease in both adult hippocampal DG and forebrain SVZ neurogenesis with brain regional differences in the AIE response that persist into adulthood.

## Introduction

Adult neurogenesis involves the formation of new brain neurons that alter neurocircuits and contribute to neuroplasticity. Adult brain neurons are generated from neural stem cells and progenitor cells in two specific neurogenic niches of the brain: the hippocampal dentate gyrus (DG) subgranular zone and the lateral ventricle subventricular zone (SVZ; Eriksson et al., 1998; Spalding et al., 2013; Ernst et al., 2014). Neurons born in the hippocampal DG differentiate and integrate into existing networks as granule cells, and neurons born in the SVZ migrate to the olfactory bulbs where they become periglomerular neurons. Neurogenesis is a complex developmental process that includes neuroprogenitor cell (NPC) proliferation, migration, differentiation and survival. Emerging new findings have found that the progenitors and developmental milestones of neurogenesis in the DG and SVZ are different (Kempermann et al., 2004, 2015; Abrous et al., 2005; Ming and Song, 2005, 2011; Lledo et al., 2006; Song et al., 2016). In the DG, six stages identified by morphology and expression of markers have been defined (Kempermann et al., 2015); whereas in the SVZ progenitor stages of differentiation have been divided into five phases of adult neurogenesis (Ming and Song, 2011). In the DG, markers of NPCs that distinguish stages of neurogenesis, including sex determining regions Y-box 2 (SOX2) which is expressed in non-radial and horizontal type-1 cells, and Tbr2, a T-box transcription factor expressed by type-2a and -2b intermediate NPCs that persist to maturation into neuroblasts (type-3). Immature/mature neuron markers include doublecortin (DCX), a microtubule-associated protein expressed in migrating neuroblasts but lost after maturation to mature granule cells in DG (Brown et al., 2003; Rao and Shetty, 2004; Couillard-Despres et al., 2005), prospero homeobox protein 1 (Prox1), which is expressed in the neuroblast, immature and mature neurons (Lavado and Oliver, 2007; Lavado et al., 2010; Hodge and Hevner, 2011), and parvalbumin (PV), which labels mature γ-aminobutyric acid (GABAergic) interneurons of the nervous system. Adult SVZ neurogenesis involves different NPCs and development (Ming and Song, 2005, 2011), briefly, radial glia-like cells (type B) in the SVZ have SOX2, Mash1 is expressed in amplifying NPC (type C), and formation of neuroblasts. Dlx2 and DCX are markers of SVZ neuroblasts and immature neurons, and mature interneurons are expressed by NeuN. These NPC maturation and markers studies provide opportunities to determine sensitivity of various NPC progenitor stages in both DG and SVZ to environment, drugs and/or other factors.

New emerging evidence has suggested that altered neurogenesis and gliogenesis may contribute to drug taking and drug seeking (Mandyam and Koob, 2012). Further, studies suggest that drug-induced loss of neurogenesis contributes to addiction and recovery from a variety of drugs, including alcohol (Mandyam and Koob, 2012; Staples and Mandyam, 2016). Previous studies have found that chronic ethanol treatment of adult rats reduces NPC proliferation in the DG (Nixon and Crews, 2002; Herrera et al., 2003; He et al., 2005) and SVZ (Crews et al., 2004; Hansson et al., 2010; Campbell et al., 2014). A binge model of alcohol dependence found reduced adult hippocampal DG neurogenesis following ethanol treatment (Nixon and Crews, 2004; Hansson et al., 2010). Interestingly, abstinence after adult binge ethanol treatment results in bursts of NPC proliferation over the first week of abstinence that restore DG neurogenesis after about 30 days of abstinence (Nixon and Crews, 2004; Nixon et al., 2008). Further, stress and chronic ethanol self-administration by adult mice reduce hippocampal neurogenesis and induce depression-like behavior that are reversed by the antidepressant fluoxetine (Stevenson et al., 2009). Although hippocampal DG neurogenesis has been linked to drug dependence, SVZ neurogenesis has not been extensively studied. One study using a rat 7-week ethanol treatment model of chronic relapsing alcohol dependence that increases ethanol self-administration results in a loss of both DG and SVZ neurogenesis (Hansson et al., 2010). Interestingly, in this study DG neurogenesis recovered over weeks of abstinence similar to the binge studies noted above whereas SVZ neurogenesis was decreased after 21 days of abstinence following ethanol treatment (Hansson et al., 2010). Thus, neurogenesis in DG and SVZ differ in NPC markers, maturation and inhibition by ethanol.

Adolescence is a critical developmental stage during which brain networks that regulate cognition and emotion mature (Dahl, 2004; Crews et al., 2016). Binge drinking is also common in human adolescents (Wechsler et al., 1995; O’Malley et al., 1998). Human and animal studies suggest that alcohol exposure during adolescence alters brain development causing unique long lasting changes in adulthood (Spear, 2000). Previous studies have found hippocampal neurogenesis is greater in adolescents than adults (He and Crews, 2007) and adolescent rat hippocampal DG neurogenesis is more sensitive to ethanol inhibition than adult DG neurogenesis (Crews et al., 2006). More recent studies using adolescent intermittent ethanol (AIE) exposure have found reduced adult neurogenesis using ethanol vapor (Ehlers et al., 2013a), intragastric (i.g) ethanol exposure (Ehlers et al., 2013a; Broadwater et al., 2014; Vetreno and Crews, 2015), intraperitoneal (i.p) exposure (Sakharkar et al., 2016) or ethanol self-administration (Briones and Woods, 2013). Broadwater et al. (2014) directly compared adolescent and adulthood binge ethanol exposure on adult neurogenesis and found AIE persistently reduced adult DG neurogenesis whereas identical adult treatment only transiently reduced DG neurogenesis suggesting ethanol exposure during adolescence, but not adulthood, markedly reduced DCX expression in the hippocampal DG 4 weeks after the final ethanol exposure. Although acute adolescent binge ethanol exposure has been found to reduce both DG and SVZ neurogenesis (Crews et al., 2006), to our knowledge the impact of AIE on proliferation and maturation of NPC in SVZ neurogenesis has not been determined. This study reports for the first time the impact of AIE exposure, a model of underage binge drinking, on multiple markers across the stages of neurogenesis in both the hippocampal DG and forebrain SVZ in adulthood. We report here that AIE causes a persistent loss of both SVZ and DG neurogenesis, and that the AIE-induced changes in markers of NPC stages and cell death suggest that the mechanisms of the AIE-induced loss of new neuron formation in DG and SVZ is different for each brain region.

## Materials and Methods

### Animals and AIE Exposure

Timed-pregnant Wistar rats (*n* = 10), young mothers at the same age, were ordered from Harlan Laboratories, Inc. (Indianapolis, IN, USA) under a protocol approved by the Institutional Animal Care and Use Committees at the University of North Carolina. All animals were maintained at 22°C under a 12:12-h light/dark cycles with free access to food and water. Timed-pregnant dams at embryonic day 17 (E17), were allowed to acclimate to our vivarium. On the day following birth (postnatal day 1, P1), litters were culled to 10 pups. On weaning at P21, male offspring were pair-housed with a same-gender, same-age non-littermate and body weight match assigned to two experimental groups, including control and AIE group. This study was done in all males. The AIE group was exposed intermittently (e.g., 2 days on, 2 days off) with ethanol (5 g/kg, 25% ethanol w/v, i.g., 10 ml/kg) during adolescence (P25–P54); and the control group was administered with the same volume of water as described previously (Liu and Crews, 2015). Body weight was measured every 4 days. All control and AIE rats grew normally from P25 (control: 44.0 ± 1.3 g; AIE: 45.9 ± 1.3 g) to P54 (control: 262.7 ± 3.9 g; AIE: 258.4 ± 4.8 g) during AIE exposure. As expected, there was a significant main difference of age with body weight [*F*_(1,384)_ = 1298.25, *p* < 0.001], and no significant effect on group [*F*_(7,384)_ = 0.07, *p* = 0.786], age × group interaction [*F*_(7,384)_ = 0.60, *p* = 0.753]. From P57 to P95 after final ethanol exposure, the body weight of all animals also revealed a significant main difference of age [*F*_(9,320)_ = 118.28, *p* < 0.001], and but no group effect [*F*_(1,320)_ = 0.01, *p* = 0.932] or age × group interaction [*F*_(9,320)_ = 0.26, *p* = 0.985]. All rats appeared normal throughout the experiment consistent with AIE binge exposure not causing observable changes in health. During AIE exposure, tail blood samples were collected and measured twice 1 h after ethanol exposure (5 g/kg i.g.) at P38 and 54. Blood ethanol concentrations (O’Malley et al., 1998) were measured using a GM7 Analyzer (Analox, London, UK). Their average values were 152.17 ± 11.09 at P38 and 211.86 ± 19.26 mg/dl at P54. Statistics showed that the BECs had a significant difference between at postnatal day 38 and 54 [*F*_(1,50)_ = 7.21, *p* < 0.01]. At P54, animals of both control and AIE group were randomly reassigned to three groups respectively with body weight match after the last ethanol exposure. BrdU (5′-bromo-2-deoxyuridine, Sigma; St. Louis, MO, USA) is incorporated into DNA during cell division. To assess proliferation, control and AIE groups were sacrificed on P57, 2 h after BrdU injection (*n* = 8/each group, 300 mg/kg, dissolved in 0.9% saline, i.p.). A second group of control and AIE rats were sacrificed on P95 2 h after BrdU injection(*n* = 8/each group, 300 mg/kg i.p.) to assess persistent changes in progenitor proliferation. For progenitor cell survival and differentiation study, a third group of control (*n* = 8) and AIE (*n* = 10) were administered BrdU 150 mg/kg daily starting at P54 for 14 days, and were allowed to survive 4 weeks after BrdU treatment, and sacrificed on P95 (See Supplementary Figure S1 for additional details).

### Animal Tissue Collection, Preparation and Immunohistochemistry

Rats were deeply anesthetized with an overdose of sodium pentobarbital, and transcardially perfused with 0.1 M phosphate-buffered saline (PBS, pH7.4) followed by 4% paraformaldehyde (in 0.1 M phosphate buffer, pH 7.4). Brains were removed, and post-fixed for 24 h in 4% paraformaldehyde at 4°C. Coronal sections were obtained at a thickness of 40 μm in 1:12 series after cryoprotection with 20% and 30% sucrose, gradually. Every 12th section was used for each of the following antigens.

For BrdU staining, DNA denaturation was performed by incubating the section in 2 N HCl for 30 min 37°C (Kuhn et al., 1996). The sections were then incubated with 0.6% hydrogen peroxide (H_2_O_2_) for 30 min to remove endogenous peroxidase activity. Anti-mouse BrdU (Chemicon, MAB3424, Temecula, CA, USA) was used at a dilution of 1:2000 and incubated overnight at 4°C, and sections were incubated with biotinylated horse anti-mouse secondary antibody (1:200, Vector Laboratories, Burlingame, CA, USA) at room temperature for 1 h. Then avidin-biotin-peroxidase complex (ABC Elite Kit, Vector Laboratories) was added for 1 h at room temperature. Finally, the BrdU-positive cells were visualized using DAB (nickel-enhanced diaminobenzidine). For all other antigens, sections were incubated in 0.6% H_2_O_2_ for 30 min, and blocked in 3% goat serum (0.1% Triton X-100) for 1 h at room temperature. In the current studies, all other primary antibodies were used with different dilutions (Table 1) overnight at 4°C. At the second day, sections were rinsed in PBS, and incubated with biotinylated secondary goat anti-rabbit or anti-mouse or anti-chicken antibody (1:200, Vector Laboratories, Burlingame, CA, USA) for 1 h at room temperature. Subsequently, the positive cells were visualized using DAB.

**Table 1:** Primary antibodies in the present study.

Antibodies	Isotype	Source/Purification	Dilution	Source
BrdU	Mouse IgG	Monoclonal	1:2000	Chemicon, Temecula, CA, USA (MAB3424)
Ki67	Rabbit IgG	Polyclonal	1:400	Abcam Inc., Cambridge, MA, USA (ab66155)
Tbr2/EOMES	Chicken IgG	Polyclonal	1:200	Lifespan Biosciences, Inc., Seattle, WA, USA (LSC123463/31819)
Sox2	Rabbit IgG	Polyclonal	1:1000	Abcam Inc., Cambridge, MA, USA (ab97959)
DCX	Rabbit IgG	Polyclonal	1:1000	Abcam Inc., Cambridge, MA, USA (ab18723)
Prox1	Rabbit IgG	Polyclonal	1:1000	Abcam Inc., Cambridge, MA, USA (ab37128)
Parvalbumin	Rabbit IgG	Polyclonal	1:1000	Abcam Inc., Cambridge, MA, USA (ab11427)
NeuN	Rabbit IgG	Polyclonal	1:1000	EMD Millipore, Billerica, MA, USA (ABN78)
Iba1	Rabbit IgG	Polyclonal	1:500	Wako/Fisher, Cambridge, MA, USA (019-19741)
Nestin	Rabbit IgG	Polyclonal	1:400	Abcam Inc., Cambridge, MA, USA (ab27952)
Mash1	Rabbit IgG	Polyclonal	1:50	Novus Biologicals, Littleton, CO, USA (NBP1-51269)
Dlx2	Rabbit IgG	Polyclonal	1:1000	Abcam Inc., Cambridge, MA, USA (ab18188)
β-2 microglobulin	Rabbit IgG	Polyclonal	1:50	Santa Cruz Biotechnology Inc., Dallas, TX, USA (sc-15366)
Cleaved caspase-3	Rabbit IgG	Polyclonal	1:1200	Cell Signaling Technology, Danvers, MA, USA (#9661)

Double fluorescence staining for BrdU and Ki67 was done using a mix of mouse anti-BrdU (1:2000, MAB3424, Chemicom, Temecula, CA, USA) and rabbit anti-Ki67 (1:400, ab66155, Abcam, Inc., Cambridge, MA, USA). Then, sections were incubated in the dark for 1 h with following secondary antibodies (1:200, a mix of goat anti-mouse 594 and anti-rabbit 488, Alexa Fluors, Molecular Probes Eugene, OR, USA). For mouse anti-BrdU (1:2000, MAB3424, Chemicon) and chicken anti-Tbr2 (1:800, S-C123463/31819, Lifespan bioscience Inc., Seattle, WA, USA), the secondary antibodies were used with a mix of goat anti-mouse 488 and anti-chicken 594 (1:200, Alexa Fluors, Molecular Probes Eugene, OR, USA). For mouse anti-BrdU (1:2000, MAB3424, Chemicom) and rabbit anti-Iba1 (1:500, 019-19741, Wako/Fisher, Cambridge, MA, USA), the secondary antibodies were used with a mix of goat anti-mouse 488 and anti-rabbit 594 (1:200, Alexa Fluors, Molecular Probes Eugene, OR, USA). For mouse anti-BrdU (1:2000, MAB3424, Chemicom) and anti-rabbit NeuN (1:1000, EMB Millipore, ABN78, Billerica, MA, USA), sections were incubated in the dark for 1 h with following secondary antibodies (1:200, a mix of goat anti-mouse 594 and anti-rabbit 488 Alexa Fluors, Molecular Probes Eugene, OR, USA). All sections were covered slips with anti-fade mounting reagent (ProLong, Molecular Probes).

### Quantification

Bioquant Nova Advanced Image Analysis (R&M Biometric, Nashville, TN, USA) was used for image capture and analysis (Crews et al., 2004). Images were captured by using an Olympus BX50 Microscope and Sony DXC-390 video camera linked to a computer. In the hippocampal DG, positive cells (BrdU+, Ki67+, Tbr2+, SOX2+, Prox1+, PV+, β-2 microglobulin+ and activated caspase-3+IR) were counted using prolife counting in the granule cell layer of the dorsal DG of HIP (Bregma from −2.30 mm to −4.52 mm; Paxinos and Watson, 1997) and expressed as cells per square millimeter with both sides of 3–5 section per animals, and the average value per mm^2^ was used (See Supplementary Figure S1D). For DCX+IR, the granule cell layer of the hippocampal DG was outlined and pixel density was measured for the outlined area (pixels/mm^2^). In the SVZ, positive cells (Nestin+, Dlx2+, BrdU+ and activated caspase-3+IR) were counted using prolife counting similar to that described by Kuhn et al. (1996) and Crews et al. (2006) in the special regions interested and expressed as cells per square millimeter with both sides of 3–5 section per animals, and the average value per mm^2^ was used. For SOX2+, Mash1+ and DCX+IR, pixel densities were measured for the outlined area (pixels/mm^2^; see Supplementary Figure S1D). In the SVZ, Bregma from 1.20 mm to −0.30 mm (Paxinos and Watson, 1997), positive immunoreactivities were measured at series of three 50 μm boxes along the length of SVZ as described (Kuhn et al., 1996; Crews et al., 2006).

Confocal analyses were conducted using LesicaSP2 AOBS Upright Laser Scanning Confocal in Michael Hooker Microscopy Facility of University of North Carolina. In the granule cell layer of hippocampal DG, 50–100 BrdU positive cells per sample were analyzed for co-labeling with Tbr2 or Iba1 or NeuN. The percentages of co-label in BrdU+IR were calculated.

### Statistical Analysis

All values (including body weight, positive cells and pixel values) were reported as mean ± SEM and analyzed using analysis of variance (ANOVA) (IBM SPSS Statistics 22). The change of body weight was analyzed using a mixed ANOVA (group × day) with day as the repeated measure. Significant effects of AIE exposure on the body weight at different days were used with Student’s *t*-test. For the effect of AIE exposure on the expression of positive cells or pixel values, ANOVA was used to test statistical significance, and followed by comparison of each group mean with Independent-Samples *T*-Test, and the *p*-value was used for statistical significance. The significant difference were considered if *p* < 0.05 at least. In this study, the percentage of the maturational decline between P57 and P95 with marker+IR expression was (P57 − P95)/P57 * 100; and the maturational increase between P57 and P95 with marker+IR expression was (P95 − P57)/P57 * 100. The percentage of AIE-induced decrease was (Control − AIE)/Control * 100; and AIE-induced increase was (AIE − Control)/Control * 100. Pearson correlations were used to determine the correlation across all markers in either the hippocampal DG or SVZ region.

## Results

### AIE Exposure Reduces Hippocampal Dentate Gyrus Neurogenesis

**Figure 1:**
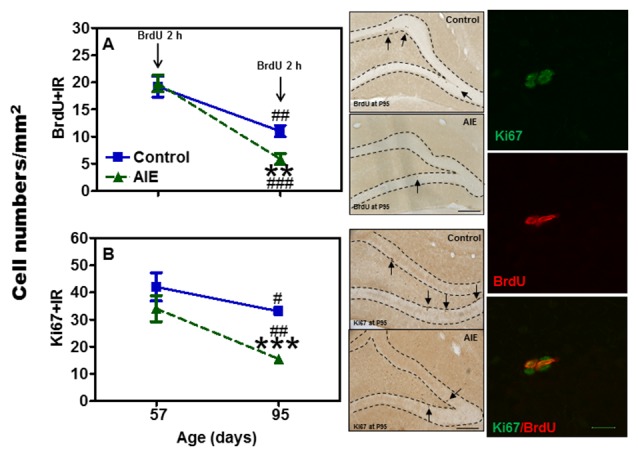
Effects of adolescent intermittent ethanol (AIE, 5 g/kg, i.g., 2 days on/2 days off) exposure on BrdU+ (2 h, **A**) and Ki67+IR **(B)** expression in the hippocampal dentate gyrus (DG) of male rat brain at P57 and P95. Figure on left side: AIE exposure remarkably decreased BrdU+ (47%, **A**) and Ki67+IR (46%, **B**) expression at P95, but not at P57. **p < 0.01, ***p < 0.001 compared with control group. BrdU+ **(A)** and Ki67+IR **(B)** expression had the maturational decline in both control (BrdU+IR: 43%; Ki67+IR: 42%) and AIE group (BrdU+IR: 70%; Ki67+IR: 63%) from P57 to P95. ^#^*p* < 0.05, ^##^*p* < 0.01, ^###^*p* < 0.001 compared with control or AIE group at P57, respectively. The data were expressed as the numbers of BrdU+ **(A)** or Ki67+IR **(B)** positive cells, each point is mean ± SEM per mm^2^ (*n* = 7~8/group). Middle panels: BrdU+ and Ki67+IR expression in the granular cell layer of the hippocampal DG at P95 (Immunohistochemical staining, Bar scale = 100 μm). Right panels: photomicrographs of confocal images in the granular cell layer of hippocampal DG, Ki67+ (green) and BrdU+ (red), Bar scale = 15 μm.

Neurogenesis in adults involves NPC proliferation followed by maturation to an integrated functional neuron over a period of several weeks. We used multiple markers of NPCs that distinguish stages of neurogenesis and neuronal maturation on P57 (3 days after last ethanol exposure) and P95 (41 days after abstinence). We first determined proliferation using two indices, Ki67 and BrdU. Ki67, an endogenous marker of cell proliferation, is a nuclear protein expressed in all phases of the cell cycle, except the resting phase. We administered BrdU 2 h before sacrifice, which marks dividing cells during S-phase of the mitotic process. We found that AIE exposure did not affect BrdU+ and Ki67+IR expression 3 days after last ethanol exposure (P57), however, 41 days later (P95) there was a difference. Control levels of both BrdU+IR (42.6 ± 5.4%) [*F*_(1,27)_ = 69.31, *p* < 0.001] and Ki67+ (42.3 ± 5.4%) [*F*_(1,27)_ = 42.39, *p* < 0.001] declined with age from P57 to P95 (Figure 1), consistent with the known maturational decline in hippocampal neurogenesis. Interestingly, the maturational decline appeared to be accelerated in AIE (BrdU: 70.4 ± 5.0%, *p* < 0.001, Ki67: 63.3 ± 3.1%, *p* < 0.001) treated animals since at P95. AIE exposure decreased BrdU+ (46.8 ± 9.0%, *p* < 0.01, Figure 1A) and Ki67+IR (46.0 ± 4.6%, *p* < 0.001, Figure 1B) in the DG compared to control P95. For Ki67+IR study, two-way ANOVA showed that there was a main significant effect of group [*F*_(1,27)_ = 10.59, *p* < 0.01]. These findings suggest AIE exposure alters the maturation of proliferating NPCs in the DG.

**Figure 2:**
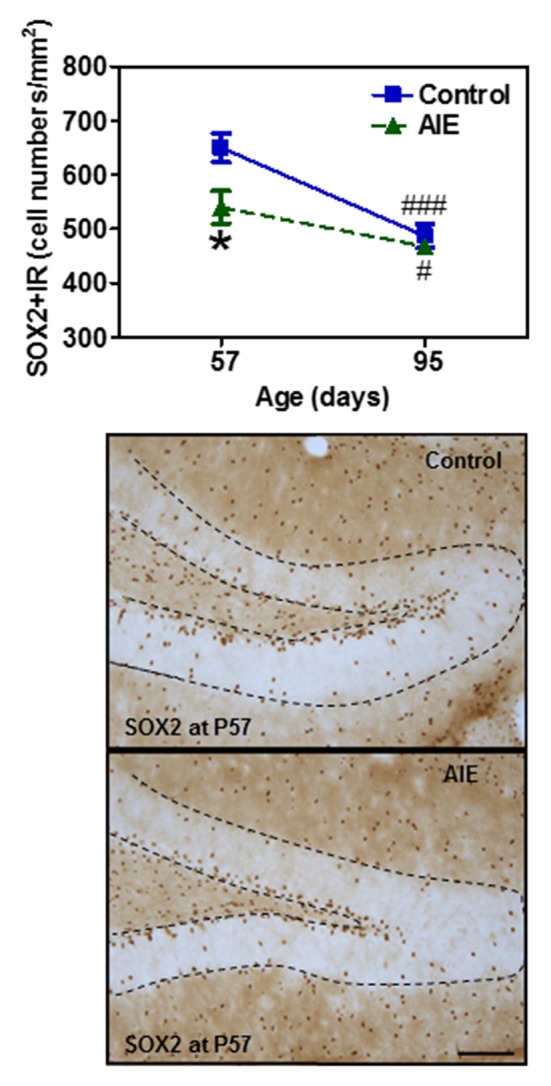
Effects of AIE (5 g/kg, i.g., 2 days on/2 days off) exposure on SOX2+IR expression in the hippocampal DG of male rat brain at P57 and P95. Figure on the top: AIE exposure remarkably decreased SOX2+IR (17%) expression at P57, but not at P95. *p < 0.05 compared with control group. SOX2+IR expression had the maturational decline in both control (28%) and AIE group (13%) from P57 to P95. ^#^*p* < 0.05, ^###^*p* < 0.001 compared with control or AIE group at P57, respectively. The data were expressed as the numbers of SOX2+IR positive cells, each point is mean ± SEM per mm^2^ (*n* = 7~8/group). Below panels: SOX2+IR expression in the granular cell layer of the hippocampal DG at P57 (Immunohistochemical staining, Bar scale = 50 μm).

**Figure 3:**
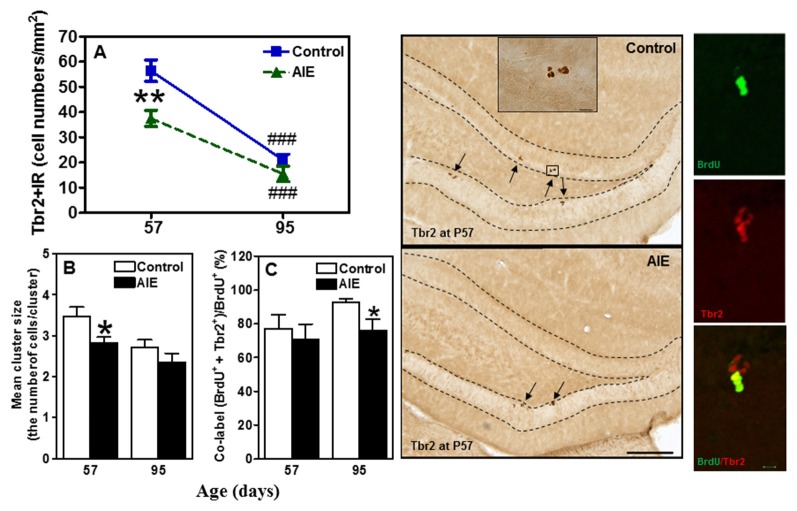
Effects of AIE (5 g/kg, i.g., 2 days on/2 days off) exposure on Tbr2+IR expression in the hippocampal DG of male rat brain at P57 and P95. **(A)** AIE exposure remarkably decreased 34% Tbr2+IR expression at P57, but not at P95. **p < 0.01 compared with control group. Tbr2+IR expression had the maturational decline in both control (63%) and AIE group (59%) from P57 to P95. ^###^*p* < 0.001 compared with control or AIE group at P57, respectively. The data were expressed as the numbers of Tbr2+IR positive cells, each point is mean ± SEM per mm^2^ (*n* = 7~8/group). **(B)** Tbr2+IR cell clusters and cell numbers per cluster were counted in the granular cell layer of the DG of control and AIE group at P57 and P95, and mean cell numbers per cluster were used. The mean cluster size of AIE group were significantly smaller than control group at P57 (*p < 0.05). **(C)** AIE exposure resulted in a reduction of co-localization of Tbr2/BrdU+ cells in BrdU+ cells compared with control group (*p < 0.05) at P95. Middle panels: Tbr2+IR expression in the granular cell layer of the hippocampal DG at P57 (Immunohistochemical staining, Bar scale = 100 μm, inset 10 μm). Right panels: photomicrographs of confocal images in the granular cell layer of the hippocampal DG, BrdU+ (green) and Tbr2+ (red), Bar scale = 10 μm.

To assess milestones of neurogenesis stages, we determined SOX2 and Tbr2 that mark NPCs of radial glial-like (Type-1) and intermediate progenitor cells (Type-2). Interestingly, both SOX2+ (Figure 2) and Tbr2+IR (Figure 3) show significant AIE-induced changes on P57 that are resolved by P95. AIE exposure significantly reduced SOX2+IR expression at P57 (16.9 ± 4.6%, *p* < 0.05, Figure 2), but not at P95. Both control and AIE group showed the maturational decline between P57 and P95 with SOX2+IR expression (control: 27.9 ± 3.4%, *p* < 0.001; AIE: 13.4 ± 1.9%, *p* < 0.01). Two-way ANOVA indicated that there was a main significant effect of group [*F*_(1,27)_ = 5.14, *p* < 0.05], age [*F*_(1,27)_ = 35.99, *p* < 0.001] and group × age interaction [*F*_(1,27)_ = 4.87, *p* < 0.05] on SOX2+IR. However, the maturational decline in AIE group was less than in control (*p* < 0.01, from P57 to P95). We determined both Tbr2+IR expression and cellular cluster size. AIE exposure significantly reduced Tbr2+IR expression (33.5 ± 4.9%, *p* < 0.01, Figure 3A) on P57, but not at P95. There was maturational decline of Tbr2+IR expression in control (62.5 ± 3.0%, *p* < 0.001) and AIE (58.5 ± 6.0%, *p* < 0.001). Two-way ANOVA indicated that there was a main significant effect of group [*F*_(1,27)_ = 23.72, *p* < 0.01], age [*F*_(1,27)_ = 129.95, *p* < 0.0001), and group × age interaction [*F*_(1,27)_ = 7.04, *p* < 0.05] on Tbr2+IR (Figure 3). Determination of mean cluster size of Tbr2+IR cells in the AIE animals (2.8 ± 0.2 cell numbers/cluster) were significantly smaller than controls (3.5 ± 0.2 cell numbers/cluster) at P57 (*p* < 0.05), however, they were not different on P95 (Control 2.7 ± 0.2 vs. AIE 2.4 ± 0.2 cell numbers/cluster, Figure 3B). Tbr2+/BrdU+ co-localization in controls was 77.3 ± 8.2% on P57 and 92.6 ± 2.2% on P95, with AIE animals having slightly fewer co-localized cells compared to control group at P95 (18.7 ± 5.8%, *p* < 0.05). Tbr2+/BrdU+ co-localization in AIE group was 70.9 ± 8.9% on P57 and 76.1 ± 6.0% on P95 (Figure 3C). These results suggest that AIE exposure reduces P57 radial glia-like and intermediate neuronal progenitor cells altering maturation of hippocampal DG.

**Figure 4:**
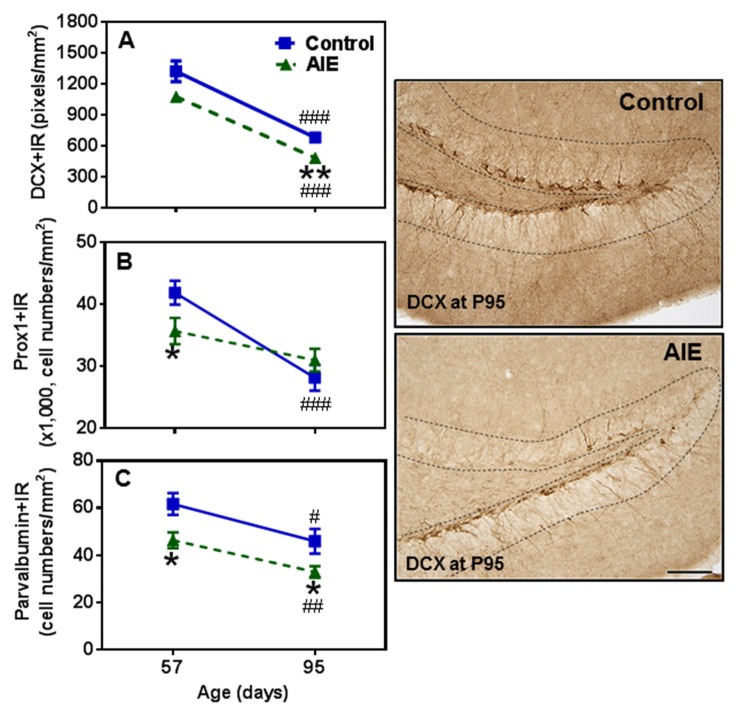
Effects of AIE (5 g/kg, i.g., 2 days on/2 days off) exposure on DCX+ **(A)** Prox1+ **(B)** and parvalbumin (PV)+IR **(C)** expression in the hippocampal DG of male rat brain at P57 and P95. AIE exposure remarkably reduced Prox1+ (**B**, 15%) and PV+IR (**C**, 27%) expression at P57 compared with control group, and reduced DCX+ (**A**, 29%) and PV+IR (**C**, 28%) expression at P95. *p < 0.05, **p < 0.01 compared with control group. There was a significantly maturational decline with DCX+ (**A**, control: 49%; AIE: 56%), Prox1+ (**B**, control: 33%) or PV+IR (**C**, control: 29%; AIE: 29%) expression in control or AIE group. ^#^*p* < 0.05, ^##^*p* < 0.01, ^###^*p* < 0.001 compared with control or AIE group at P57, respectively. The data were expressed as pixels of DCX+ **(A)** or the numbers of Prox1+ **(B)** or PV+IR **(C)** positive cells, each point is mean ± SEM per mm^2^ (*n* = 7~8/group). Right panels: DCX+IR expression in the granular cell layer of the hippocampal DG (Immunohistochemical staining, Bar scale = 50 μm).

To determine immature neuron formation, we used DCX and Prox1 markers as well as the mature GABAergic marker PV. All three markers decreased with age between P57 and P95 in the control group, DCX+ (48.6 ± 3.9%, *p* < 0.001, Figure 4A), Prox1+ (32.9 ± 5.0%, *p* < 0.001, Figure 4B) and PV+IR (28.7 ± 5.9%, *p* < 0.05, Figure 4C) consistent with the maturational decline in DG neurogenesis. AIE exposure resulted in a reduction with Prox1+ (15.3 ± 4.8%, *p* < 0.05) and PV+IR expression (26.8 ± 6.0%, *p* < 0.05), and had a reductive trend in DCX+IR (*t* = 2.069, *p* = 0.059) compared with P57 controls. At P95, AIE exposure reduced DCX+ (29.1 ± 5.1%, *p* < 0.01) and PV+IR (28.2 ± 5.1%, *p* < 0.05), but not Prox1+IR (Figure 4). These findings indicate a persistent loss of DCX+IR immature neurons and PV GABAergic neurons in adults following AIE exposure.

### Survival and Differentiation of NPCs in Hippocampal Dentate Gyrus after AIE Exposure

**Figure 5:**
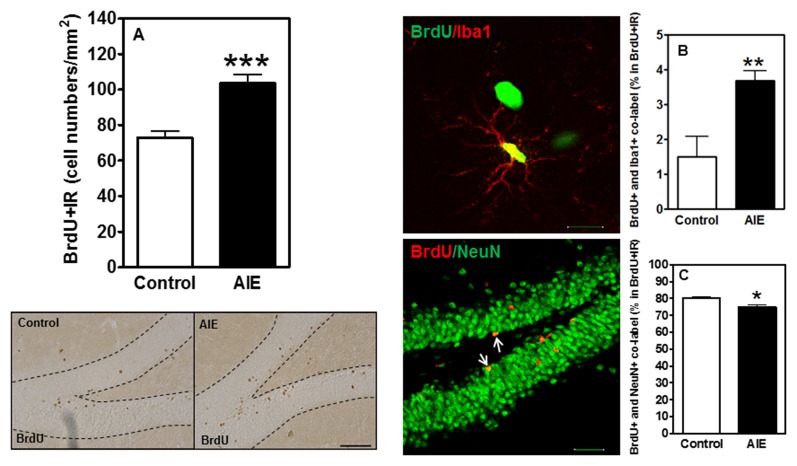
Effects of AIE (5 g/kg, i.g., 2 days on/2 days off) exposure on survival BrdU+IR expression after abstinence in the hippocampal DG of male rat brain at P95. **(A)** BrdU+IR expression was measured after 6 weeks of abstinence, 4 weeks following labeling with BrdU (150 mg/kg, i.p. 14 days). AIE exposure significantly increased BrdU+IR expression in the DG **(A)** ***p < 0.001 compared with control group. The data were expressed as the numbers of BrdU+IR positive cells, each point is mean ± SEM per mm^2^ (*n* = 8~10/group). Left below panels: BrdU+IR expression in the granular cell layer of the hippocampal DG (Immunohistochemical staining, Bar scale = 50 μm). Middle panels show photomicrographs of confocal images in the granular cell layer of the hippocampal DG, top one: BrdU+ (green) and Iba1+ (red) (Bar scale = 15 μm), and bottom one: BrdU+ (red) and NeuN+ (green) (Bar scale = 50 μm). **(B)** A small percentage of BrdU+ cells were positive Iba1+ 1.5% in control vs. 3.7% in AIE (**p < 0.01). **(C)** 74% BrdU+ co-labeled with NeuN+ in AIE was less than in control group (80%, *p < 0.05).

To investigate the survival and differentiation of neurogenesis, BrdU was injected daily for 14 days to label dividing NPCs (P54-P68) and rats sacrificed weeks later allowing BrdU+ NPCs to mature. Surprisingly, we found that AIE exposure remarkably increased BrdU+IR expression 42.11 ± 6.92% [*F*_(1,16)_ = 21.40, *p* < 0.001, Figure 5A]. Co-localization studies indicated that BrdU+ co-labeled with mature neuronal marker NeuN+ was 80.1 ± 1.1% in controls that was lower in the AIE group (74.4 ± 1.3%, *p* < 0.05, Figure 5C), suggesting slightly fewer neurons after AIE exposure. Interestingly, a small percentage of BrdU+ cells positive for microglial Iba1+ was increased by AIE from a control level of 1.5 ± 0.6% to 3.7 ± 0.3%, (*p* < 0.01) in AIE treated rats (Figure 5B). These findings suggest an altered differentiation of NPCs in hippocampal DG following AIE exposure.

### AIE Exposure Increases β-2 Microglobulin and Activated Caspase-3 Expression in the Hippocampal Dentate Gyrus

**Figure 6:**
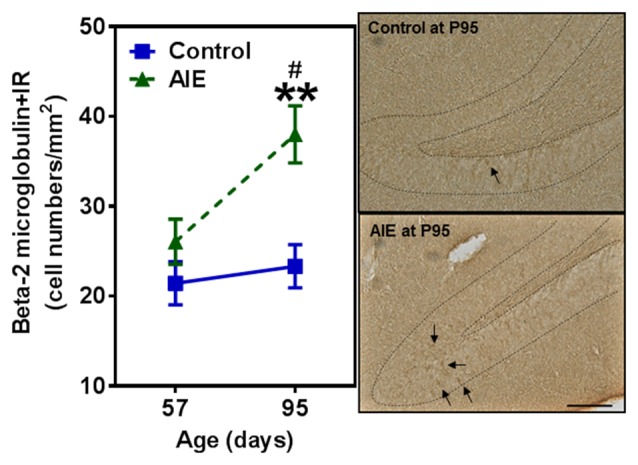
Effects of AIE (5 g/kg, i.g., 2 days on/2 days off) exposure on β2M +IR expression in the hippocampal DG of male rat brain at P57 and P95. Figure on the left: AIE exposure remarkably increased β2M+IR expression (63%) compared with control group at P95. **p < 0.01 compared with control group. β2M+IR expression had a significantly maturational increase in AIE group (47%). ^#^*p* < 0.05 compared with AIE group at P57. The data were expressed as the numbers of β2M+IR positive cells, each point is mean ± SEM per mm^2^ (*n* = 7~8/group). Right panels: β2M+IR expression in the granular cell layer of the hippocampal DG (Immunohistochemical staining, Bar scale = 50 μm).

**Figure 7:**
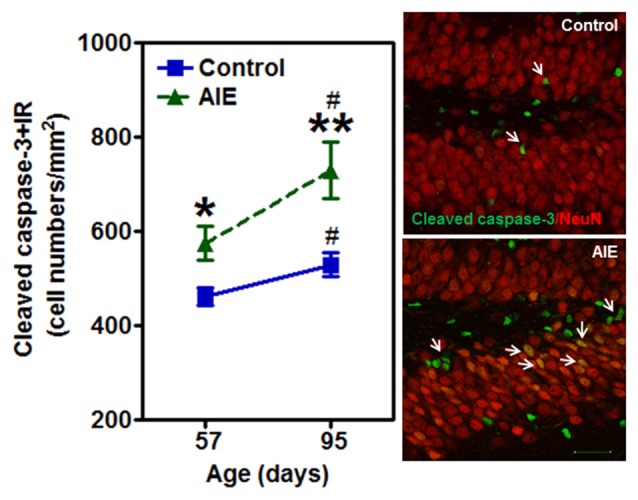
Effects of AIE (5 g/kg, i.g., 2 days on/2 days off) exposure on activated caspase-3+IR expression in the hippocampal DG of male rat brain at P57 and P95. Figure on the left: AIE exposure remarkably increased the apoptosis marker activated caspase-3+IR from P57 (27%) to P95 (41%) compared with control group. *p < 0.05, **p < 0.01 compared with control group. Activated caspase-3+IR expression had a significantly maturational increase in control (16%) and AIE group (29%). ^#^*p* < 0.05 compared with control or AIE group at P57, respectively. The data were expressed as the numbers of activated caspase-3+IR positive cells, each point is mean ± SEM per mm^2^ (*n* = 8/group). Right panels: photomicrographs of confocal images in the granular cell layer of the hippocampal DG, activated caspased-3+ (green) and NeuN+ (red), Bar scale = 30 μm.

To investigate mechanisms, we assessed immune signaling with β-2 microglobulin (β2M) and cell death using activated caspase-3 (as cleaved caspase-3). β2M is a major histocompatability complex 1 (MHC1) protein that is expressed on microglia and neurons, is involved in synaptic stripping and is known to inhibit hippocampal neurogenesis (Smith et al., 2015; Yousef et al., 2015). Caspase-3 is a protease which is activated during cell death, particularly apoptosis, and an antibody recognizes the active protease providing a marker of dying cells. Previous studies have found that injection of β2M into young adult mice results in loss of DG Tbr2+ and DCX+ NPCs (Smith et al., 2015), similar to our finding in young adults following AIE exposure. We found that AIE exposure did not alter DG β2M+IR expression at P57, whereas AIE-treated animals had about double the number of cells expressing β2M+IR at P95 (Figure 6). AIE-treated animals showed maturational increase expression from P57 to P95 (46.6 ± 11.6%, *p* < 0.05), not in control group. Two-way ANOVA of β2M+IR cells showed that there was a main effect of group [*F*_(1,27)_ = 13.27, *p* < 0.001] and age [*F*_(1,27)_ = 6.82, *p* < 0.05], but a trend effect of group × age interaction [*F*_(1,27)_ = 3.59, *p* = 0.069]. Interestingly, activated caspase-3+IR expression was slightly increased by AIE at P57 (26.7 ± 8.7%, *p* < 0.05). Maturation of controls to P95 slightly increased activated caspase-3+IR, but the increase of AIE group at P95 was about double the level of controls (Figure 7), e.g., increases between P57 and P95 for controls is 15.5 ± 4.4%, (*p* < 0.05) compared with AIE increases of 29.2 ± 7.0%, (*p* < 0.05). At P95, activated caspase-3+IR in DG after AIE exposure was increased 41.2 ± 8.5%, (*p* < 0.01) compared with controls (Figure 7). Two-way ANOVA of activated caspase-3+IR cells indicated that there was a main effect of group [*F*_(1,28)_ = 16.88, *p* < 0.0001] and age [*F*_(1,28)_ = 8.54, *p* < 0.01], but no effect of group × age interaction [*F*_(1,28)_ = 1.29, *p* = 0.266]. Interestingly, the AIE-induced increase in activated caspase-3+IR positively correlated with the AIE-induced reduction of Ki67+IR at P57 [*F*_(1,5)_ = 11.5, *r* = 0.83, *p* = 0.020], consistent with increased cell death reducing proliferating NPCs (Table 2). Similarly, in adulthood at P95 the AIE-induced increase in activated caspase-3+IR positively correlated with the AIE-induced reduction DCX+IR [*F*_(1,6)_ = 8.3, *r* = 0.76, *p* = 0.028] and Prox1+IR [*F*_(1,6)_ = 7.5, *r* = 0.75, *p* = 0.034], consistent with AIE-induced increases in cell death contributing to loss of adult neurogenesis (Table 2). Thus, DG cell death and expression of β2M+IR is increased by AIE exposure and appears to contribute to the loss of both proliferating NPCs and maturing neuron loss in adulthood.

**Table 2:** Pearson correlations across markers in the hippocampal dentate gyrus (DG) and subventricular zone (SVZ) of male rat brain.

Markers	Markers in the DG			Markers in the SVZ	
	Ki67	DCX	Prox1	SOX2	Dlx2
BrdU	-	*F*_(1,5)_ = 11.2, *r* = 0.83 *p* = 0.020 (AIE, P57)	-	-	*F*_(1,5)_ = 9.9, *r* = 0.82 *p* = 0.025 (Control, P95)
		-	*F*_(1,6)_ = 10.0, *r* = 0.79	-	-	-
		*p* = 0.020 (AIE, P95)			
SOX2	*F*_(1,5)_ = 13.3, *r* = 0.85 *p* = 0.015 (AIE, P57)	-	-	N/A	-
DCX	-	N/A	-	-	*F*_(1,6)_ = 6.9, *r* = 0.73 *p* = 0.04 (AIE, P95)
Activated caspase-3	*F*_(1,5)_ = 11.5, *r* = 0.83, *p* = 0.020 (AIE, P57)	-	-	*F*_(1,6)_ = 14.9, *r* = 0.85 *p* = 0.008 (AIE, P95)	-
		-	*F*_(1,6)_ = 8.3, *r* = 0.76 *p* = 0.028 (AIE, P95)	*F*_(1,6)_ = 7.5, *r* = 0.75 *p* = 0.034 (AIE, P95)	-	-

*AIE (5 g/kg, i.g., 2 days on/2 days off) group at P57 and P95 with significant F, r and *p* values. Only correlation of *p* < 0.05, *p* < 0.01 are shown. (-), no any correlation*.

### Subventricular Zone Neurogenesis and AIE Exposure

**Figure 8:**
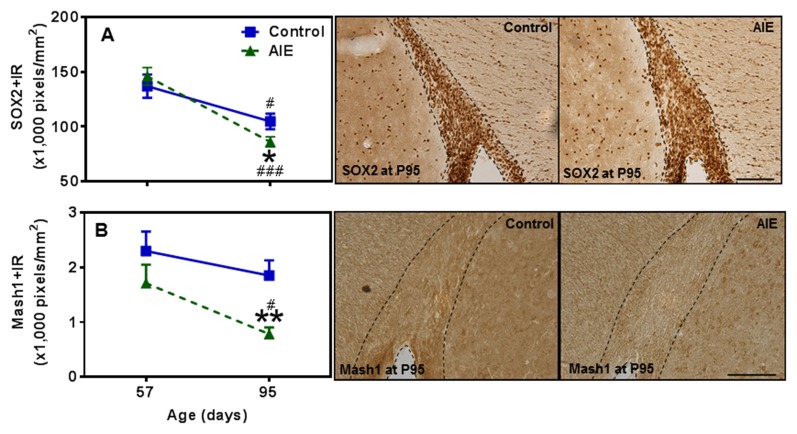
Effects of AIE (5 g/kg, i.g., 2 days on/2 days off) exposure on SOX2+ and Mash1+IR expression in the subventricular zone (SVZ) of male rat brain at P57 and P95. AIE exposure remarkably decreased SOX2+ (18%, **A**) and Mash1+IR (59%, **B**) at P95 compared with control group. *p < 0.05, **p < 0.01 compared with control group. There was a significant maturational decline with SOX2+ (**A**, control: 25%; AIE: 41%), and Mash1+IR (**B**, AIE: 56%) expression in control or AIE group. ^#^*p* < 0.05, ^###^*p* < 0.001 compared with control or AIE group at P57, respectively. The data were expressed as pixels of SOX2+ and Mash1+IR, each point is mean ± SEM per mm^2^ (*n* = 7~8/group). Right panels: SOX2+ and Mash1+IR expression in the SVZ at P95 (Immunohistochemical staining, Bar scale = 100 μm on SOX2+IR; 50 μm on Mash1+IR).

SVZ and hippocampal DG differ in adult neuroprogenitors and neurogenesis. SOX2+ and Mash1+IR mark SVZ early progenitors, and both showed a maturational decline between P57 and P95 (Figure 8). SOX2+IR did not differ between groups at P57, but the maturational decline in controls was 24.5 ± 4.5% (*p* < 0.05) compared to the AIE decline of 41.1 ± 3.2% (*p* < 0.001) that resulted in reduced expression of SOX2+IR at P95 (18.3 ± 4.1%, *p* < 0.05, Figure 8A). Two-way ANOVA revealed that there was a main effect of age [*F*_(1,26)_ = 33.92, *p* < 0.0001], without effect of group [*F*_(1,26)_ = 0.37, *p* = 0.549], group × age interaction [*F*_(1,26)_ = 3.05, *p* = 0.092] on SOX2+IR. Similarly, Mash1+IR was not significantly decreased by AIE at P57, but by P95 AIE exposure decreased Mash1+IR to 59.2 ± 6.3% (*p* < 0.05, Figure 8B) of control. Two-way ANOVA showed that there was a significant effect of age [*F*_(1,27)_ = 6.02, *p* < 0.05], group [*F*_(1,27)_ = 8.81, *p* < 0.01], without effect of group × age interaction [*F*_(1,27)_ = 0.79, *p* = 0.382] on Mash1+IR. Interestingly, no correlation of SVZ markers was found at P57 in either controls or AIE exposure animals (Table 2). Determination of BrdU+IR 2 h. after injection (S-phase marker of cell proliferation) did not find a change in SVZ (Table 3). Also nestin+IR (a marker of neural stem cell, type B) expression in the SVZ at P57 and P95 were not altered by AIE exposure (Table 3). These finding suggest SVZ neurogenesis does not show immediate changes following AIE exposure, but following maturation to adulthood at P95 led to an AIE induced loss of early progenitor markers Mash1 and SOX2 in the SVZ.

**Table 3:** Adolescent intermittent ethanol (AIE, 5 g/kg, i.g., 2 days on/2 days off) exposure did not impact the expression of BrdU+ (2 h after single injection), nestin+, β-2 microglobulin+ and activated caspase-3+IR expression in the SVZ of male rat brain at P57 and P95.

Age (day)	Group	BrdU + IR (2 h) (cell number × 1000/mm^2^)	Nestin + IR (cell number × 1000/mm^2^)	β-2 microglobulin + IR (cell number × 1000/mm^2^)	Activated caspase-3 + IR (cell number × 1000/mm^2^)
57	Control	1.90 ± 0.11	23.87 ± 1.12	2.81 ± 0.50	6.68 ± 0.41
	AIE	2.04 ± 0.13	22.24 ± 0.57	2.63 ± 0.27	6.36 ± 0.28
95	Control	1.95 ± 0.13	23.58 ± 0.72	4.01 ± 0.37	6.61 ± 0.38
	AIE	1.80 ± 0.16	23.33 ± 0.76	4.47 ± 0.034^##^	6.63 ± 0.33

*^##^p < 0.01, compared to AIE group at P57*.

**Figure 9:**
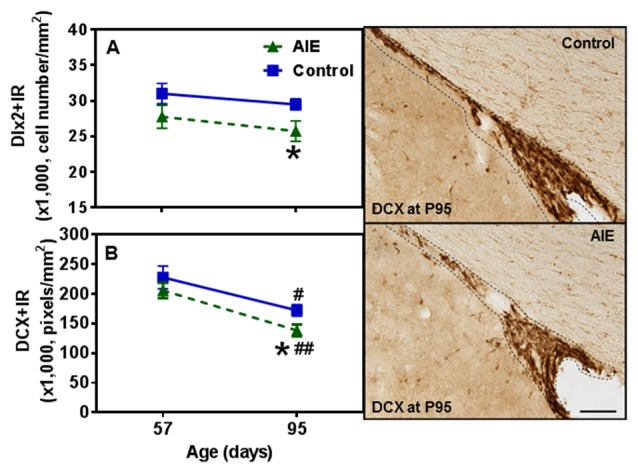
Effects of AIE (5 g/kg, i.g., 2 days on/2 days off) exposure on Dlx2+ and DCX+IR expression in the SVZ of male rat brain at P57 and P95. Figure on the left: AIE remarkably decreased Dlx2+ (13%, **A**) and DCX+IR (20%, **B**) at P95 compared with control group. *p < 0.05 compared with control group. There was a significant maturational decline of DCX+IR (control: 26%; AIE: 33%) in control and AIE group. ^#^*p* < 0.05, ^##^*p* < 0.01 compared with control or AIE group at P57, respectively. The data were expressed as the cell numbers of Dlx2+IR, and pixels of DCX+IR, each point is mean ± SEM per mm^2^ (*n* = 7~8/group). Right panels: DCX+IR expression in the SVZ at P95 (Immunohistochemical staining, Bar scale = 100 μm).

To further investigate SVZ neurogenesis, we determined neuroprogenitor markers Dlx2+IR and DCX+IR as well as NPC survival using BrdU+IR. Similar to the other SVZ markers studied, AIE exposure did not markedly alter neurogenesis markers at P57, just after AIE exposure, but after maturation to P95 AIE exposure was found to significantly reduce Dlx2+IR (12.7 ± 4.9% *p* < 0.05) and DCX+IR (19.6 ± 5.8%, *p* < 0.05) expression compared with controls (Figures 9A,B). Dlx2+IR did not show a maturational decline, but DCX+IR had maturational decline between P57 and P95 in the control (25.5 ± 5.0%, *p* < 0.05) and AIE group (32.7 ± 4.9%, *p* < 0.01) in the SVZ (Figure 9B), respectively. Two-way ANOVA revealed that there was a main effect of age [*F*_(1,27)_ = 1.73, *p* = 0.199], without effect of group [*F*_(1,27)_ = 6.73, *p* = 0.015] and group × age interaction [*F*_(1,27)_ = 0.04, *p* = 0.853] on Dlx2+IR. And there was a significant effect of age [*F*_(1,24)_ = 20.19, *p* < 0.0001], group [*F*_(1,24)_ = 4.19, *p* = 0.05], without effect of group × age interaction [*F*_(1,24)_ = 0.19, *p* = 0.671] on DCX+IR. The AIE-induced decrease in Dlx2+ and DCX+IR at P95 correlated across individuals (Table 2). SVZ progenitor survival was determined following repeated BrdU (150 mg/kg i.p.) daily injected at P54 followed by weeks of maturation to P95 as described above. We found that AIE exposure markedly decreased SVZ BrdU+IR expression 41 days after abstinence in the SVZ (20.5 ± 2.8%, *p* < 0.05). One-way ANOVA revealed that there was significant effect of group [*F*_(1,16)_ = 9.25, *p* < 0.01] (see Supplementary Figure S2). Although these findings indicate AIE exposure caused a loss of adult SVZ neurogenesis, unlike hippocampus, we did not find a change in β2M+ and activated caspase-3+IR expression in the SVZ compared with controls at P57 or P95 (Table 3). These findings indicate that AIE exposure alters SVZ leading to a progressive persistent loss of SVZ neurogenesis that appears to increase with age.

## Discussion

**Figure 10:**
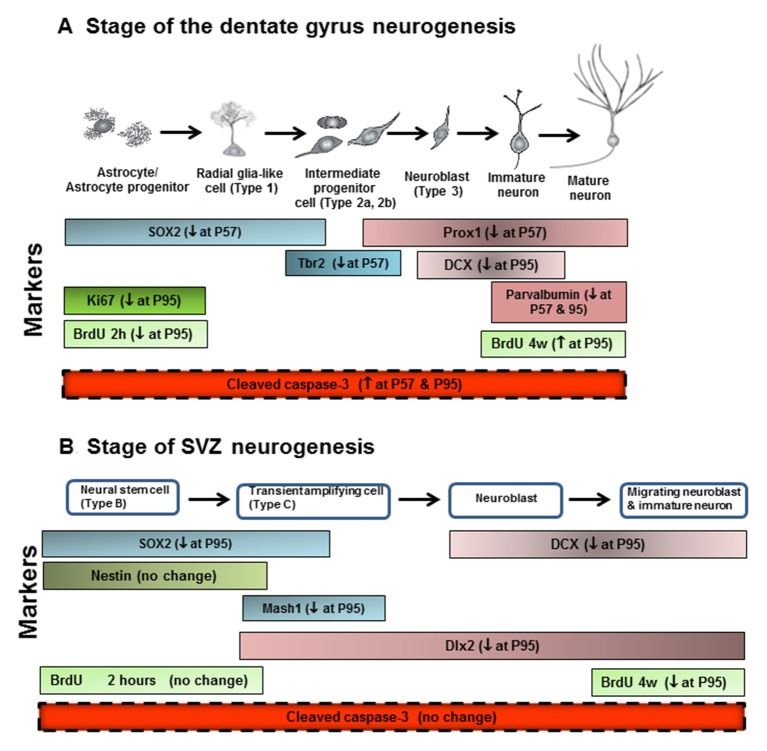
Neurogenesis stages in the DG of the adult hippocampus and SVZ of lateral ventricle. **(A)** Schematic diagram representing the progression of neuronal development (Bonaguidi et al., 2012), and specific markers in the different developmental stages of the adult DG, such SOX2, Tbr2, Prox1, DCX and PV. Ki67 protein is present during all active phases of the cell cycle (G_1_, S, G_2_, and mitosis), but is absent from resting cells (G_0_). Bromodeoxyuridine (BrdU), used in the detection of proliferating cells in living tissues as exogenous marker, is incorporated into the newly synthesized DNA of replicating cells (during the S phase of the cell cycle). For the proliferation study, animals were sacrificed 2 h after injecting BrdU with 300 mg/kg (i.p.). For the survival study, animals were sacrificed 4 weeks after injecting BrdU with 150 mg/kg (i.p.) for 14 days. **(B)** Schematic diagram representing the progression of neuronal development, and specific markers in the different developmental stages of the adult SVZ, such as SOX2, nestin, Mash1, DCX and Dlx2. ↓ at P57, decreased at postnatal day 57; ↓ at P95, decreased at postnatal day 95; ↑ at P57, increased at postnatal days 57; ↑ at P95, increased at postnatal days 95.

In this study we investigated the impact of AIE exposure, a model of human adolescent binge drinking, on hippocampal DG and SVZ neurogenesis. Neurogenesis is a dynamic self-renewal multistage process that involves the proliferation, migration, differentiation, and functional integration of new neurons into the preexisting neural circuitry that persists into adulthood in these two brain regions. We report here that AIE exposure decreases hippocampal progenitor makers SOX2+, Ki67+, Tbr2+ and BrdU+IR proliferation as well as markers of neuronal maturation, e.g., neurogenesis markers DCX+, Prox1+ and PV+IR (Figure 10). These finding are consistent with previous studies of hippocampal neurogenesis that AIE vapor treatment reduces adult Ki67+, DCX+ and BrdU+IR survival markers of neurogenesis in association with increased disinhibitory behavior and increased activated caspase-3+IR (Ehlers et al., 2013a). Similarly, AIE treatment with i.p injection of ethanol has been found to decrease adult hippocampal Ki67+ and DCX+IR while increasing HDAC activity and decreasing BDNF in association with increased anxiety (Sakharkar et al., 2016). Previous studies have found adolescents are more sensitive to ethanol inhibition of neurogenesis than adults (Crews et al., 2006). Studies by Nixon’s group have investigated a continuous intoxication 4 day binge alcohol dependence model in adolescence and reported decreased hippocampal NPC survival 28 days after ethanol exposure, increased markers of cell death, and reduced DCX+IR, but no change in hippocampal Ki67+IR just after alcohol exposure ended (Morris et al., 2010; McClain et al., 2011) similar to our findings just after AIE exposure at P57. They have found that adolescent binge alcohol exposure inhibits neurogenesis through alterations in NPC cell cycle kinetics, especially decreasing the proportion of NPCs in S-phase. Further, the 4 day binge model found reduced SOX2+IR did not persist following adolescent alcohol exposure (McClain et al., 2011) similar to our finding of SOX2+IR returning to control levels at P95, in adulthood. Overall, all studies agree adolescent ethanol exposure inhibits hippocampal neurogenesis through multiple mechanisms including increases in cell death. Recent studies have found that the AIE-induced persistent loss of neurogenesis that is not found following identical treatment in adults (Broadwater et al., 2014). The AIE studies presented here add maturational changes and new progenitor stage specific markers (Lindsey and Tropepe, 2006; Kempermann et al., 2015). Assessment of NPC proliferation markers in hippocampal DG, e.g., Ki67+ and BrdU+IR at 2 h, indicates AIE exposure accelerate the maturational declines in NPC proliferation between P57, late adolescence, and young adulthood (P95). In contrast, early neuroprogenitor transcription factor markers of radial-glial and intermediate neuronal progenitors, e.g., SOX-2+ and Tbr2+IR, respectively, are decreased after AIE exposure (P57), but decline less during abstinent maturation to P95, consistent with AIE exposure insulting early progenitors that are maturing. Conditional null mutant Sox2 mice have reduced neurogenesis, stem/precursor cells and a smaller hippocampus in adulthood consistent with Sox2 and neurogenesis being required for normal hippocampal maturation (Favaro et al., 2009). Similarly, previous studies indicate Tbr2 is a transcription factor uniquely expressed in DG NPCs that regulates progenitor cell fate in adult DG that is lost as neurons mature (Hodge et al., 2008, 2012). Prox1 is also a marker of the type-2 intermediate progenitors (Tbr2+ cells) in the DG (Urbán and Guillemot, 2014). AIE exposure resulted in the reduction of SOX2+, Tbr2+ and Prox1+IR expression in the DG at P57, suggesting that AIE-reduced radial glia and intermediate progenitor cells mainly happened after last ethanol exposure, but not on 41 days after abstinence in the hippocampal DG. Although AIE exposure causes an overall reduction in adult neurogenesis, the pools of SOX2+, Tbr2+ and Prox1+IR cells return to control levels in young adulthood (P95). Co-localized Tbr2+ and BrdU+ staining was reduced at P95 following AIE exposure consistent with fewer NPCs in S phase of cell cycle and a reduced rate of NPC proliferation and/or increased cell death. A reduced rate of progenitor proliferation is consistent with the reduced Ki67+ and BrdU+IR at 2 h, found after AIE exposure at P95. Further, we find AIE exposure increased activated caspase-3+IR cells at P57 that was further increased at P95. We also find increased activated caspase-3+IR at P57 correlates with decreased Ki67+IR in hippocampus DG consistent with increased cell death in early progenitors just after AIE exposure. In AIE exposed young adults at P95, activated caspase-3+IR cells only correlate with DCX+ cells suggesting increased cell death is an important mechanism contributing to the persistent loss of neurogenesis following AIE.

The mechanisms of persistent loss of neurogenesis following AIE exposure may be related to increased expression of proinflammatory cytokines and other innate immune signaling molecules. Previous studies have found that AIE exposure induces multiple proinflammatory cytokines, Toll-like receptors (TLRs) and RAGE, as well as HMGB1 an agonist at these immune receptors (Vetreno and Crews, 2012, 2014; Vetreno et al., 2013), that persists after AIE exposure and increases during maturation to young adulthood (Vetreno and Crews, 2012). Similarly, another study found that AIE exposure reduced adult hippocampal neurogenesis is associated with increases in adult hippocampal expression of the proinflammatory cytokines TNFα and MCP1 as well as the protease MMP9 and innate immune transcription factor NFκB (Vetreno and Crews, 2014). Further, this study found that treatment with lipopolysaccharide, a TLR4 agonist known to induce brain cytokines (Qin et al., 2007), reduces adult hippocampal neurogenesis (Vetreno and Crews, 2014). Another related finding is that AIE exposure reduces hippocampal BDNF expression (Sakharkar et al., 2016) suggesting loss of trophic support with increased neuroimmune gene expression after AIE exposure. β2M is a MHC1 protein that is expressed on monocytes and other cells as well as on brain microglia and neurons, where it is involved in synaptic stripping and is known to inhibit hippocampal neurogenesis (Smith et al., 2015; Yousef et al., 2015). In the current study, we found a marked increase in β2M+IR cells at P95, but not just after AIE exposure at P57, suggesting a progressive induction in expression of neuroimmune signaling β2M+IR cells. Proinflammatory cytokines (Fan and Pang, 2017) and TLRs (Rolls et al., 2007) can inhibit neurogenesis through increased progenitor death and/or increased differentiation to glia. Interestingly, β2M+IR expression similar to the receptor RAGE+IR (Vetreno and Crews, 2012; Vetreno et al., 2013) is not induced just after AIE treatment, but is triggered by AIE exposure and induced during maturation to young adulthood. Our study focused on adolescent maturation to adulthood and found increased β2M+IR expression and the greatest reductions in neurogenesis in young adulthood. Previous studies have reported that age-related increases in the DG β2M cause the age-related loss of neurogenesis during senescence (Smith et al., 2015; Yousef et al., 2015). Our findings are consistent with AIE-induced β2M+IR reflecting a progressive increase in neuroimmune signaling that reduces neurogenesis through increased cell death of hippocampal neuroprogenitors.

In the present study, we add new data on the impact of AIE on SVZ neurogenesis (Figure 10). SVZ NPCs are different from hippocampal NPCs (Kriegstein and Alvarez-Buylla, 2009; Ming and Song, 2011). In the adult mammalian brain, new neurons are generated in the SVZ and migrate through the white matter into the neocortex (Gould et al., 1999). Neurogenesis in adult SVZ is found along the walls of the lateral ventricles and has stages of adult SVZ neurogenesis that differ from DG (Ming and Song, 2005). Proliferating radial glia-like SOX2+ cells (type B cells), give rise to transient amplifying neuroblasts and immature Mash1+ neuroprogenitors (type C cells) that migrate through the rostral migratory stream (RMS). Hansson et al. (2010) in an adult rat 7 weeks chronic relapsing alcoholic model found that DCX+IR and SOX2+IR in the SVZ was reduced just after treatment and the reduced SVZ neurogenesis persisted for 21 days of abstinence. Other studies in adult mice have reported weeks of ethanol self-administration reduce SVZ BrdU+ cells (Campbell et al., 2014). We previously found that acute ethanol dose-dependently decreased cell proliferation in the SVZ of adolescent rats (Crews et al., 2006). A previous AIE exposure study in Sprague-Dawley rats that were sacrificed on P74, 26 days after the last ethanol exposure, found decreased hippocampal and SVZ neurogenesis with age, similar to that reported here, as well as the persistent loss in adulthood, although in SVZ the AIE reduction was not statistically significant at P74 (Broadwater et al., 2014), consistent with our finding of the AIE reduced SVZ neurogenesis increasing with age and being significant at P95. We studied AIE-treated rats 41 days after the last AIE exposure and found a significant, persistent loss of adult neurogenesis as indicated by decreased SOX2+, Mash1+, DCX+ and Dlx2+ cells in the SVZ. This is consistent with an emerging loss of SVZ neurogenesis during age-related maturation that requires time, e.g., at P95 we report here a significant loss of SVZ neurogenesis, but our studies reported here at P57 and Broadwater et al. (2014) at P74 found no change or a trend, respectively. Taken together, these studies suggest AIE exposure causes a progressive persistent change in the neurogenic niche of SVZ that weeks after AIE exposure ends reduces SOX2+ neural stem cells, Mash1+ progenitors, Dlx2+ neuroblasts and DCX+ immature neurons. These decreases in SVZ occur without an increase in activated caspase-3+ cells suggesting increased cell death is not a major contributor to the persistent loss of SVZ neurogenesis like it is in hippocampus DG. The AIE exposure induced loss of neurogenesis appears to be related to a progressive loss of SVZ neuroprogenitors. Mash1 is also known as achaete-scute homolog 1 (ASCL1) and is a transcription factor associated with stem cells neuronal differentiation commitment and Dlx2 is a neuroprogenitor-immature neuron transcription factor that are both decreased in young adults after AIE exposure consistent with a loss of early stem cells as well as differentiating progenitors. DCX marks immature neurons and BrdU (4 weeks) assesses neurogenesis. AIE exposure reduced both DCX+ and BrdU+IR (4 weeks) assessments indicating loss of neurogenesis. Thus, together these findings suggest AIE exposure leads to a persistent and growing loss of SVZ neuroprogenitors and neurogenesis.

Although our study did not investigate AIE-induced changes in behavior, loss of neurogenesis may contribute to AIE exposure induced behavior changes in adults. AIE exposure has been found to reduce adult cognitive flexibility and increase negative affect-anxiety (Crews et al., 2016). Loss of hippocampal neurogenesis has been proposed to reduce cognitive flexibility (Anacker and Hen, 2017) and to be associated with stress-induced depression-like behavior and the ability of anti-depressants to reverse depression. In addition, AIE-induced loss of neurogenesis has been found to correlate with loss of hippocampal volume (Ehlers et al., 2013b), a finding that mimics the reduced hippocampal volume found in human alcoholics and depressed individuals. Thus, the AIE induced loss of neurogenesis may contribute to cognitive and mood changes in adulthood that increase risks of adult psychopathology.

## Author Contributions

FTC and WL designed the study. WL performed the experiments and collected data. WL and FTC analyzed the data and wrote the manuscript. All authors read and approved the manuscript.

## Conflict of Interest Statement

The authors declare that the research was conducted in the absence of any commercial or financial relationships that could be construed as a potential conflict of interest.

## Abbreviations

AIE: adolescent intermittent ethanol
ANOVA: analysis of variance
BEC: blood ethanol concentration
DCX: doublecortin
DG: dentate gyrus
HIP: hippocampus
i.g.: intragastric
i.p.: intraperitoneal
NPCs: neuroprogenitor cells
PBS: phosphate-buffered saline
PV: parvalbumin
Prox1: prospero homeobox protein 1
RMS: rostral migratory stream
Sox2: sex determining regions Y-box 2
SVZ: subventricular zone
Tbr2: T-box brain protein 2
TLR: Toll-like receptor.
