# Supplementary material for: Persistent Decreases in Adult Subventricular and Hippocampal Neurogenesis Following Adolescent Intermittent Ethanol Exposure
Source: Front Behav Neurosci. 2017 Aug 14;11:151. doi: 10.3389/fnbeh.2017.00151 (PMC5557743; doi:10.3389/fnbeh.2017.00151)

Supplementary Figure 1: The experimental designs (A, B) and body weight (C), blood ethanol concentration (BEC, C) and the interested brain regions (D) of Wistar male rats. (A) or (B) The timeline of the experimental design, Adolescent intermittent ethanol (AIE) exposure started at postnatal days 25 (P25). Animals were intermittently exposed (e.g. 2 days on, 2 days off) with ethanol (5 g/kg, 25% ethanol w/v, i.g.) during adolescence (P25-54), control group was administered with the same volume water. At P54, animals of both control and AIE groups were randomly reassigned to three groups respectively with body weight match. (A) For the proliferating study, rats of two controls and two AIE groups were sacrificed 2 hours after BrdU (300 mg/kg i.p.) injection at P57 and P95, respectively. (B) For the survival and differentiation sutdy, the group of control and AIE was intraperitoneally administered with BrdU at a dose of 150 mg/kg daily starting at P54 for 14 days. Rats were allowed to survive for 4 weeks, and sacrificed at P95. (C) Body weight was measured every four days during experimental procedure. BEC were measured 1 hour after treating with ethanol (5 g/kg, i.g.) at P38 and P54, using a GM7 Analyser. Each point is mean ± SEM (n=50/group during P25-P54; n=34/group during P55-P95). (D) The hippocampal dentate gyrus (DG) and subventricular zone (SVZ) were studied by the immunohistological method. Rat atlas panels reprinted from Paxinos and Waston (Paxinos and Watson, 2007).


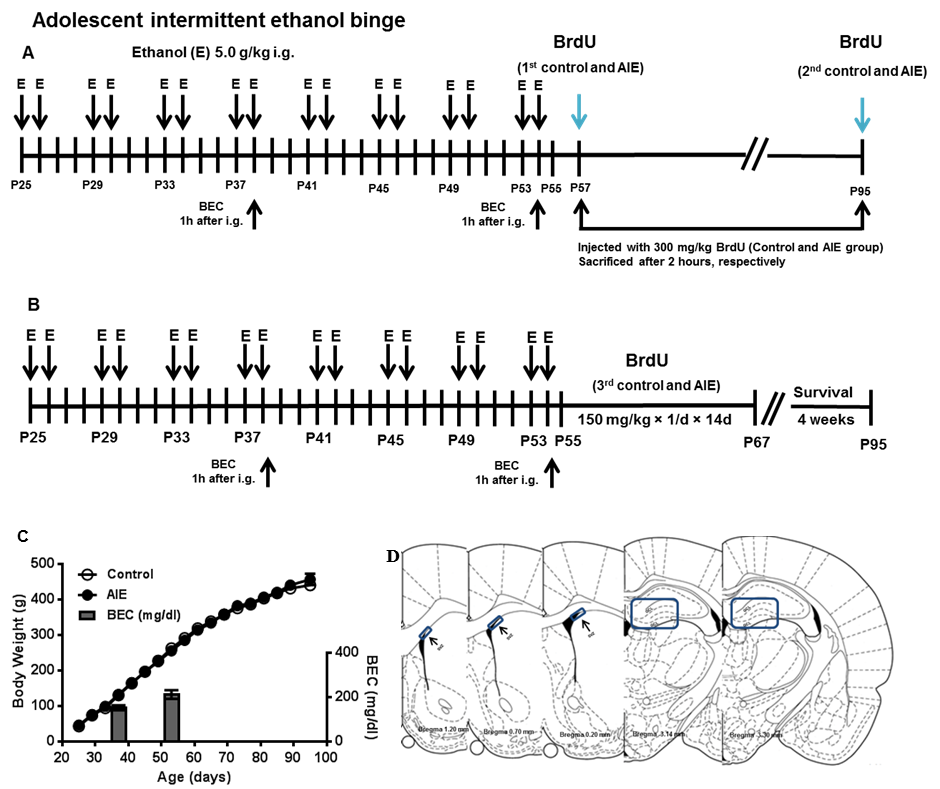

Supplement: Supplementary file 1 [file Data_Sheet_1.docx]
