# Supplementary material for: Persistent Decreases in Adult Subventricular and Hippocampal Neurogenesis Following Adolescent Intermittent Ethanol Exposure
Source: Front Behav Neurosci. 2017 Aug 14;11:151. doi: 10.3389/fnbeh.2017.00151 (PMC5557743; doi:10.3389/fnbeh.2017.00151)

Supplementary Figure 2. Effects of adolescent intermittent ethanol (AIE, 5 g/kg, i.g., 2 days on, 2 days off) exposure on survival BrdU+IR expression after abstinence in the subventricular zone of male rat brain at P95. BrdU+IR expression was measured after 6 weeks of abstinence, 4 weeks following labeling with BrdU (150 mg/kg, i.p. 2 weeks). AIE exposure significantly decreased BrdU+IR expression in the SVZ. **p*<0.05 compared with control group. The data were expressed as the numbers of BrdU+IR positive cells, each point is mean ± SEM per mm^2^ (n=8-10/group).


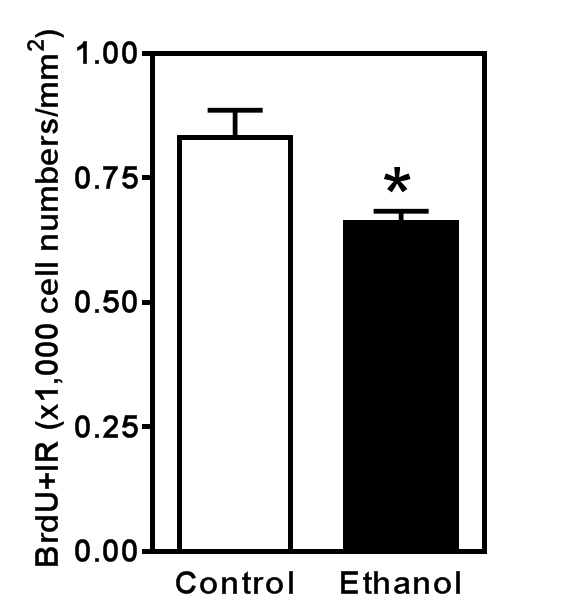

Supplement: Supplementary file 2 [file Data_Sheet_2.docx]
